# Efficacy of Early Warning Scores as the Prediction Tool for Detecting Patients With Acute Deterioration in a High Dependent Unit

**DOI:** 10.7759/cureus.71971

**Published:** 2024-10-20

**Authors:** Jun Ehara, Yohei Masuda, Koichi Hayashi, Yasuhiro Norisue, Shigeki Fujitani

**Affiliations:** 1 Department of Internal Medicine, Tokyo Bay Urayasu Ichikawa Medical Center, Urayasu, JPN; 2 Department of Pulmonary Medicine, Tokyo Bay Urayasu Ichikawa Medical Center, Urayasu, JPN; 3 Department of Emergency and Critical Care Medicine, St. Marianna University School of Medicine, Kawasaki, JPN; 4 Department of General Medicine, Okayama University Graduate School of Medicine, Dentistry and Pharmaceutical Sciences, Okayama, JPN; 5 Department of Emergency and Critical Care Medicine, Tokyo Bay Urayasu Ichikawa Medical Center, Urayasu, JPN

**Keywords:** early warning score (ews), high dependency unit, national early warning score (news), rapid response team (rrt), receiver operating characteristic (roc) analysis

## Abstract

Background

Early detection and response to patient deterioration are essential to prevent serious outcomes such as unplanned intensive care unit (ICU) transfers and cardiac arrests. Rapid response systems (RRS) have been implemented globally, leading to a reduction in in-hospital mortality. In high-dependency units (HDUs), where patient monitoring is more frequent than in general wards, but staffing levels are lower than in intensive care units (ICUs), the challenge of identifying deteriorating patients persists. The Early Warning Score System (EWSS), including the National EWS (NEWS) and the artificial intelligence (AI)-based Visensia Safety Index (VSI), offer tools for early detection of patients’ exacerbations. This study evaluates the effectiveness of NEWS and VSI in predicting patient deterioration in an HDU in Japan.

Methods

This single-center retrospective cohort study was conducted at a 344-bed acute care hospital. The study population included patients admitted to the HDU between September 27, 2019, and December 31, 2019. Study outcomes were unexpected ICU admission, endotracheal intubation, de novo use of vasopressors, de novo use of non-invasive positive pressure ventilation (NPPV), cardiac arrest, and the composite outcome (any of these outcomes). The predictive accuracy of the National Early Warning Score (NEWS) and the Visensia Safety Index (VSI) for detecting outcomes within 24 hours from each time point was analyzed.

Results

A total of 356 patients were included, with a median age of 76 years old (interquartile range (IQR): 64-84) and a median HDU stay of 2.0 days (IQR: 2.0-3.0). Among the 2648 analyzed vital sign sets, the median NEWS score was 5.0 (IQR: 3.0-7.0) and the median VSI score was 1.1 (IQR: 0.7-1.9). Twenty-six patients (7.3%) experienced outcomes. Among these, 13 (3.7%) required unexpected ICU transfers, 6 (1.7%) required endotracheal intubation, 15 (4.2%) required de novo vasopressor use, and 7 (2.0%) required de novo NPPV. NEWS could predict unexpected ICU admissions with good accuracy (the area under the curve (AUC) 0.834), as well as de novo vasopressor use (AUC 0.765) and the composite outcome (AUC 0.701). VSI also demonstrated modest predictive efficacy for unexpected ICU transfers (AUC 0.767), endotracheal intubation (AUC 0.712), and de novo vasopressor use (AUC 0.733). Based on ROC analysis, the appropriate threshold for NEWS ranged between 6.0 and 8.0, and the optimal threshold for VSI was found to be 1.5. Chronologically, NEWS remained stable before the occurrence of outcomes, whereas VSI showed a significant upward trend, suggesting that VSI may be more sensitive to detecting early deterioration.

Conclusions

In this study, both NEWS and VSI demonstrated modest accuracy in predicting unexpected ICU admission and vasopressor use in an HDU. While NEWS high risk (7≥) is an appropriate cutoff value for detecting adverse outcomes, particularly lowering the VSI cutoff to 1.5, may enhance the identification of high-risk patients.

## Introduction

Recognizing and promptly responding to patient deterioration is crucial to prevent life-threatening adverse outcomes such as unplanned intensive care unit (ICU) transfers and cardiac arrests [1]. For many years, strategies have been discussed to identify critically ill patients earlier in non-ICU settings. In recent decades, the rapid response system (RRS), designed for the timely identification and intervention of clinically deteriorating patients, has been developed and has gained widespread implementation worldwide [2]. The implementation of RRS is associated with a significant reduction in in-hospital mortality and cardiac arrests [3-5].

High-dependency units (HDUs) are intermediate care units between the general wards and ICUs. In HDUs, telemetry monitoring and close vital sign monitoring are provided for relatively sicker patients than in general wards [6,7]. However, HDUs typically have fewer medical staff compared to ICUs. In Japan, the required patient-to-nurse ratio is 4-5 to 1 in HDUs, compared to 2 to 1 in ICUs and 7 to 1 in general wards [8]. Patients in HDUs are usually managed by non-intensive care physicians such as internists or surgeons.

A single-parameter criterion (where RRS is activated if any single vital sign abnormality meets the threshold) has been conventionally used as a trigger for RRS activation. However, a single-parameter criterion may underestimate the need for RRS activation when individual vital sign values are only slightly outside the normal range [2]. The Early Warning Score System (EWSS) was developed as a more accurate trigger for RRS activation compared to the single-parameter criterion. This system assigns scores to various vital signs and mental statuses and assesses the aggregation of these scores. In 2012, the Royal College of Physicians developed a standardized EWSS for the National Health Service, known as the National EWS (NEWS) [9]. NEWS has been validated in general wards, emergency room (ER), and prehospital settings, demonstrating its utility in predicting unplanned ICU transfers, cardiac arrests, and short-term mortality within 48 hours [10-12]. The Visensia Safety Index (VSI) is another artificial intelligence (AI)-based EWSS that integrates and analyzes data from five vital signs, and it has been approved by the Food and Drug Administration (FDA) [13]. The advantage of VSI is that the score is automatically calculated from measured vital signs, allowing medical staff easy access to the net score via telemetry monitors, where alert notifications can be readily observed.

Although there is a higher risk of patient deterioration in HDUs, a method for timely detection of these deteriorating patients has not yet been fully established. Therefore, we conducted this study to investigate the efficacy of NEWS and VSI as predictive tools for detecting patient deterioration in an HDU.

## Materials and methods

Study design, population, and setting

This retrospective cohort study was conducted at a single center, a 344-bed teaching community hospital with 14 ICU beds and 12 high-dependency unit (HDU) beds. To eliminate any potential impact from the COVID-19 pandemic, we limited the study period to before the onset of the pandemic. The study population included all patients admitted to the HDU between September 27th, 2019 and December 31st, 2019. The repeated HDU admissions of the same patient were regarded as different cases. Patients who had initiated vasopressor therapy or invasive mechanical ventilation prior to the HDU admission were excluded. Conversely, patients who began non-invasive positive pressure ventilation (NPPV) either before or within one hour of the HDU admission were included. Most patients in the HDU were managed by non-intensive care physicians, such as internists or surgeons. According to the Japanese healthcare insurance system, the nurse-to-patient ratio in the HDU is 4:1, meaning one nurse cares for four patients. Vital signs were recorded at least every four hours in the HDU. At our institution, patients requiring RRS activation were assessed by a medical emergency team (MET) led by an intensivist.

Data collection

The vital signs and physiological measurements, including respiratory rate (RR), oxygen saturation (SpO_2_), body temperature (BT), blood pressure (BP), heart rate (HR), presence or absence of supplemental oxygen, and level of consciousness graded by the Glasgow Coma Scale (GCS), were retrospectively extracted from the medical records of patients admitted to the HDU during the study period. Age, sex, departments of the primary diagnosis, and outcomes were also collected. The NEWS and VSI (OBS Medical, Cirencester, UK) for the study population were retrospectively calculated. The NEWS was calculated as defined by the Royal College of Physicians. Aggregates of each NEWS were categorized into three groups: low-risk (NEWS: 0-4), intermediate-risk (NEWS: 5-6), and high-risk (NEWS: ≥7), based on the NEWS threshold criteria [9]. The VSI was categorized into four groups: normal-risk (VSI: 0-1.4), low-risk (VSI: 1.5-2.0), medium-risk (VSI: 2.1-2.9), and high-risk (VSI: ≥3), according to the OBS medical clinical user guide [14].

Outcomes

The outcomes included unexpected ICU transfers, endotracheal intubation, de novo vasopressor use, de novo NPPV use, cardiac arrest, and a composite outcome (any of these outcomes). The predictive accuracy (sensitivity, specificity, and the area under the receiver operating characteristic (ROC) curve) of NEWS and VSI for outcomes within 24 hours of each time point was analyzed. Time points with incomplete vital signs set required to calculate NEWS or VSI were excluded from the analysis. The time trends of NEWS and VSI before the first outcome were also collected.

Statistical analysis

R (The R Foundation for Statistical Computing, Vienna, Austria, version 3.3.3) and EZR (Saitama Medical Center, Jichi Medical School, Saitama, Japan, version 1.3.5) were used for data analysis. EZR is a graphical user interface for R, designed as a modified version of R Commander to incorporate statistical functions frequently used in biostatistics [15]. The Mann-Whitney U test or Kruskal-Wallis test was used to compare continuous variables between two groups or among more than three groups, respectively. Fisher’s exact test was applied to analyze categorical data. DeLong’s test was used to compare the area under two correlated ROC curves. A p-value of <0.05 was considered statistically significant. The predictive performance was evaluated using the area under the ROC curve (AUC), with AUC values categorized as follows: 0.50 to 0.60 (fail), 0.60-0.70 (poor), 0.70-0.80 (fair), 0.80-0.90 (good), and 0.90-1.00 (excellent) [16,17].

Ethical approval and consent to participate

This study adhered to the guidelines of the Declaration of Helsinki and was approved by the Institutional Review Board at Tokyo Bay Urayasu Ichikawa Medical Center (Approval Number: 841, date of approval: 28th April 2023). As this was a retrospective observational study, a waiver of informed consent was obtained.

## Results

Patient basic characteristics

**Figure 1:**
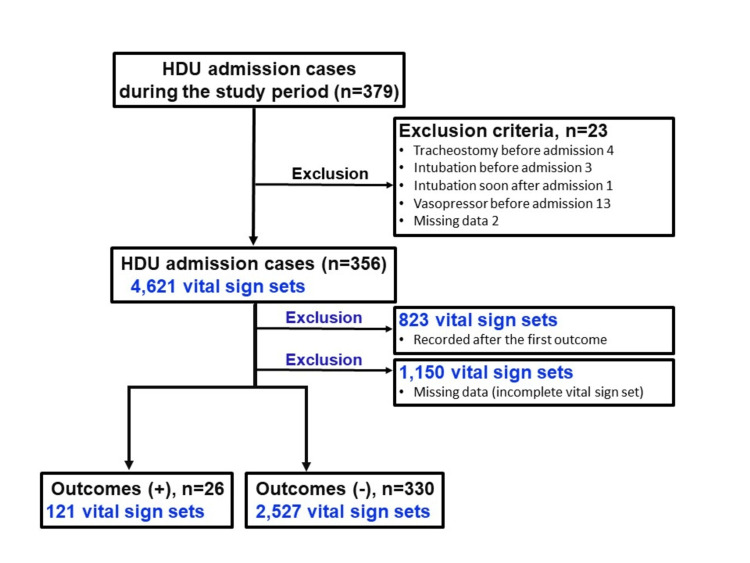
Patient and vital sign set enrolment flow chart. HDU: high-dependency unit.

During the study period, 379 admission cases were recorded in our HDU. Twenty-one cases with outcomes occurring before HDU admission and two cases with missing data were excluded, leaving a total of 356 admission cases for analysis (Figure 1). Of the 356 admissions, 22 patients were admitted twice, and two patients were admitted three times. Each instance was considered a new admission after discharge.

Table 1 presents the baseline characteristics of the patients. Of the 356 patients, 233 (65.4%) were male, with a median age of 76 years (interquartile range (IQR), 64-84 years). The median length of stay in the HDU was 2.0 days (IQR: 2.0-3.0). Two hundred twenty-seven patients (64.3%) were admitted from the ER/clinic, 64 (18.1%) from the ICU, and 60 (16.9%) from general wards. The most frequent department of primary diagnosis was the Department of Cardiology (31.3%), followed by General Internal Medicine (20.6%), Neurosurgery (14.9%), and Cardiovascular Surgery (11.5%).

**Table 1:** Patient characteristics. IQR: interquartile range, ER: emergency room, ICU: intensive care unit, NA: not acquired, NPPV: non-invasive positive pressure ventilation, HDU; high-dependency unit. The results with continuous or categorical variables were analyzed using the Mann-Whitney U test or Fisher’s exact test, respectively. A p-value of <0.05 was considered statistically significant.

	Total (n=356)	The composite outcome		p-value
		(+, n=26)	(-, n=330)	
Age, median (IQR)	76.0 (64.0-84.0)	76.0 (66.0-85.8)	76.0 (64.0-83.0)	0.841
Male/female: n (male %)	233/123 (65.4)	14/12 (53.8)	219/111 (66.4)	0.205
Length of HDU stay, median (IQR)	2 (2-3)	3 (2-4.75)	2 (2-3)	0.167
Admission: n (%)				0.01
Clinic/ER	227 (64.3)	12 (46.3)	215 (65.2)	
ICU (stepdown)	64 (18.1)	3 (11.5)	61 (18.5)	
General ward	60 (16.9)	11 (42.3)	49 (14.8)	
NA	5 (1.4)	0	5 (1.5)	
NPPV on HDU admission: n (%)	16 (4.5)	6 (23.1)	10 (3.0)	<0.001
Department: n (%)				0.561
General internal medicine	73 (20.6)	4 (15.4)	69 (20.9)	
Cardiology	111 (31.3)	7 (26.9)	104 (31.5)	
Gastroenterology	35 (9.9)	3 (11.5)	32 (9.7)	
Respiratory medicine	15 (4.2)	3 (11.5)	12 (3.6)	
Nephrology	10 (2.8)	0	10 (3.0)	
General surgery	15 (4.2)	1 (3.8)	14 (4.2)	
Cardiovascular surgery	41 (11.5)	5 (19.2)	36 (10.9)	
Neurosurgery	53 (14.9)	3 (11.5)	50 (15.2)	
Orthopedic surgery	2 (0.6)	0	2 (0.6)	

Physiological parameters and early warning scores (EWS)

Among the 356 patients, 4621 vital sign sets were collected. Of these, 823 sets were recorded after the first outcome, and 1,150 sets with missing data were excluded. This left 2648 vital sign sets for analysis (Figure 1). The median NEWS was 5.0 (IQR: 3.0-7.0), and the median VSI score was 1.1 (IQR: 0.7-1.9). Based on NEWS thresholds, the low-risk, intermediate-risk, and high-risk groups consisted of 1131 vital sign sets (42.7%), 735 vital sign sets (27.8%), and 782 vital sign sets (29.5%), respectively. Based on VSI scores, the normal-risk, low-risk, medium-risk, and high-risk groups consisted of 1650 vital sign sets (62.3%), 434 vital sign sets (16.4%), 337 vital sign sets(12.7%), and 226 vital sign sets (8.5%), respectively (Table 2). The median NEWS score for patients who experienced outcomes was significantly higher than for those who did not (8.0 (IQR: 6.0-10.0) vs. 5.0 (IQR: 3.0-7.0), p<0.001). Similarly, the median VSI score for patients with outcomes was significantly higher than for those without outcomes (1.8 (IQR: 0.9-2.6) vs. 1.1 (IQR: 0.7-1.8), p<0.001).

**Table 2:** Physiologic parameters and EWS. IQR: interquartile range, GCS: Glasgow Coma Scale, NEWS: National Early Warning Score, VSI: Visensia Safety Index. The results with continuous or categorical variables were analyzed using the Mann-Whitney U test or Fisher’s exact test, respectively. A p-value of <0.05 was considered statistically significant.

	Total (n=2648)	The composite outcome		p-value
		(+, n=121)	(-, n=2527)	
Body temperature, median (IQR)	36.8 (36.6-37.2)	36.9 (36.6-37.3)	36.8 (36.6-37.2)	0.158
Respiratory rate, median (IQR)	20 (17-23)	24 (21-29)	20 (17-23)	<0.001
Oxygen saturation, median (IQR)	96 (95-98)	95 (93-96)	96 (95-98)	<0.001
Supplemental oxygen use: n (%)	1565 (59.1)	100 (82.6)	1465 (58.0)	<0.001
Heart rate, median (IQR)	81 (71-92.3)	85 (79-94)	81 (71-92)	0.001
Systolic blood pressure, median (IQR)	126 (112-140)	121 (101-139)	126 (112-140)	0.006
Any deficit of GCS (below 14): n (%)	1292 (48.8)	79 (65.3)	1213 (48.0)	<0.001
NEWS	5.0 (3.0-7.0)	8.0 (6.0-10.0)	5.0 (3.0-7.0)	<0.001
NEWS category: n (%)				<0.001
Low risk (≤４points)	1131 (42.7)	19 (15.7)	1112 (44.0)	
Intermediate risk (5-6 points)	735 (27.8)	23 (19.0)	712 (28.2)	
High risk (7≥ points)	782 (29.5)	79 (65.3)	703 (27.8)	
VSI	1.1 (0.7-1.9)	1.8 (0.9-2.6)	1.1 (0.7-1.8)	<0.001
VSI category: n (%)				<0.001
Normal risk (≤1.4 points)	1650 (62.3)	46 (38.0)	1604 (63.5)	
Low risk (1.5-2.0 points)	434 (16.4)	24 (19.8)	410 (16.2)	
Medium risk (2.1-2.9 points)	337 (12.7)	26 (21.5)	311 (12.3)	
High risk (≥3 points)	227 (8.6)	25 (20.7)	202 (8.0)	

Outcomes and prediction efficacy

Twenty-six (7.3%) patients experienced outcomes. Among these, 13 (3.7%) required unexpected ICU transfers, 6 (1.7%) required endotracheal intubation, 15 (4.2%) required de novo vasopressor use, and 7 (2.0%) required de novo NPPV. No patient experienced cardiac arrest (Table 3).

**Table 3:** Outcomes of this study. ICU: intensive care unit, NPPV: non-invasive positive pressure ventilation.

Outcomes	Total (n=356)
Unexpected ICU transfer: n (%)	13 (3.7)
Endotracheal intubation: n (%)	6 (1.7)
De novo use of vasopressors: n (%)	15 (4.2)
De novo use of NPPV: n (%)	7 (2.0)
Cardiac arrest: n	0
Composite outcome: n (%)	26 (7.3)

**Figure 2:**
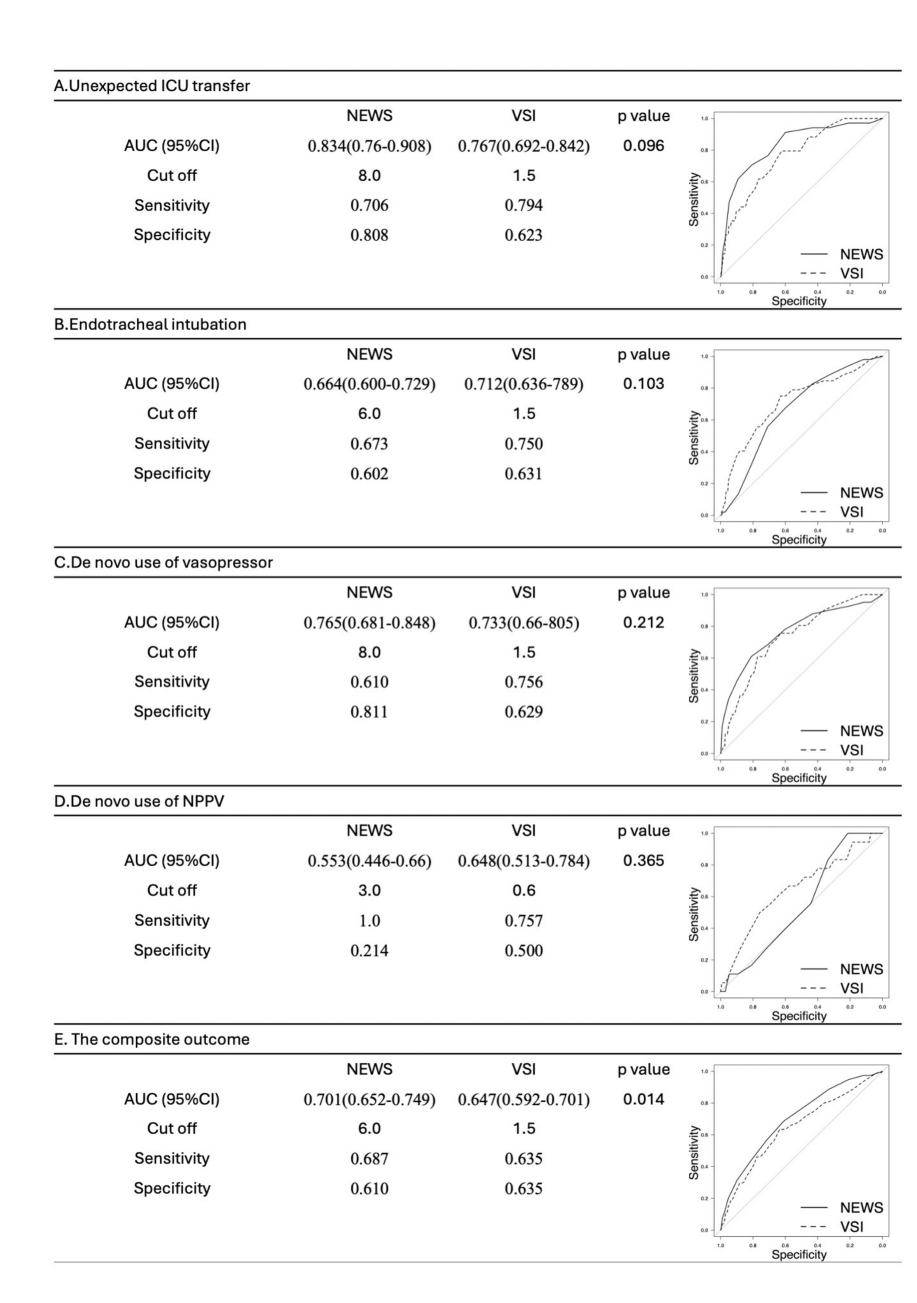
Prediction accuracies and ROC analysis of EWS on outcomes. AUC: area under the curve, CI: confidence interval, NEWS: National Early Warning Score, VSI: Visensia Safety Index, NPPV: non-invasive positive pressure ventilation. DeLong’s test was used for comparing the area under two correlated ROC curves. A p-value of <0.05 was considered statistically significant.

Figure 2 shows the ROC curves for NEWS and VSI predicting outcomes within 24 hours from each time point. The area under the curve (AUC) for NEWS predicting unexpected ICU transfer, endotracheal intubation, de novo vasopressor use, de novo NPPV use, and the composite outcome were 0.834 (95% CI: 0.760-0.908), 0.664 (95% CI: 0.600-0.729), 0.765 (95% CI: 0.681-0.848), 0.553 (95% CI: 0.446-0.660), and 0.701 (95% CI: 0.652-0.749), respectively. The AUC for VSI predicting unexpected ICU transfer, endotracheal intubation, de novo vasopressor use, and de novo NPPV, and the composite outcome use were 0.767 (95% CI: 0.692-0.842), 0.712 (95% CI: 0.636-0.789), 0.733 (95% CI: 0.660-0.805), 0.648 (95% CI: 0.513-0.784), and 0.647 (95% CI: 0.592-0.701), respectively (Figure 2). The AUC for NEWS was significantly superior to that of VSI for the composite outcome (p=0.014).

Figure 2 also displays the optimal cut-off values for NEWS and VSI, along with the corresponding sensitivity and specificity, based on the ROC curves for each outcome.

**Figure 3:**
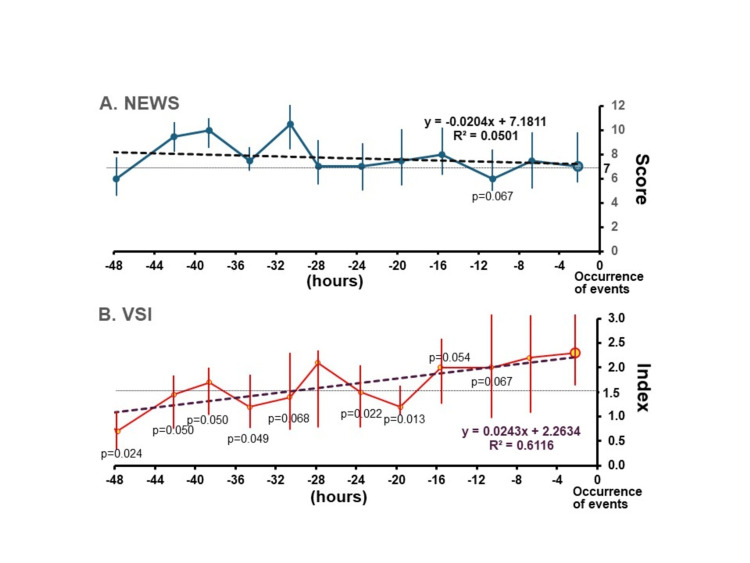
Temporal changes in early warning score before occurrence of composite events. (A) NEWS trend from 48 hours to the time of outcomes. Dotted line: Regression line for time-dependent changes in NEWS, X-axis, hours; Y-axis, NEWS. (B) VSI trend from 48 hours to the time of outcomes. Dotted line: Regression line for time-dependent changes in VSI, X-axis, hours; Y-axis, VSI. NEWS: National Early Warning Score, VSI: Visensia Safety Index. Statistical analysis was performed with the Kruskal-Wallis test followed by the Steel-Dwass test as the time point nearest the event occurrence for control. A p-value of <0.05 was considered statistically significant.

In terms of chronological data, while NEWS remained consistently elevated prior to the outcomes, VSI demonstrated a significant upward trend leading up to the composite outcome (Figure 3).

**Figure 4:**
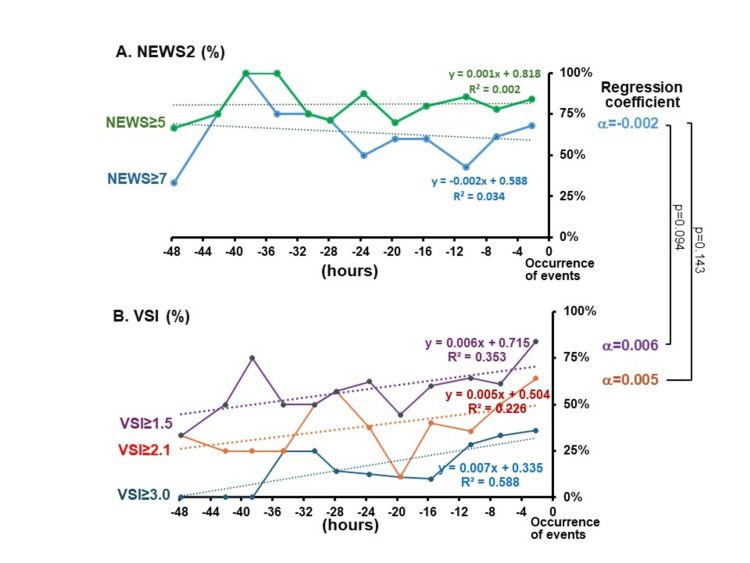
Temporal changes in proportions of patients with various values of NEWS and VSI at each time point. (A) Temporal changes in the proportions of patients with NEWS≥7 (blue) or NEWS ≥5 (green). (B) Temporal changes in the proportions of patients with VSI≥1.5 (purple), VSI≥2.1 (orange), or VSI≥3.0 (dark blue). X-axis: hours before event occurrence; y-axis: proportion of patients with various NEWS or VSI. p-values denote the differences in regression coefficient between NEWS≥7 and VSI≥2.1 or VSI≥1.5. A p-value of <0.05 was considered statistically significant. NEWS: National Early Warning Score; VSI: Visensia Safety Index.

When comparing the percentage of patients with VSI >1.5 to those with NEWS >7 using the regression coefficient (alpha), VSI showed a higher trend in the regression coefficient (p=0.094) (Figure 4).

## Discussion

To the best of our knowledge, this is the first study to evaluate the efficacy of NEWS and VSI as predictive tools for detecting clinical deterioration in HDU patients. The severity of illness in HDU patients is generally higher than in general wards, making it critical to promptly detect any signs of deterioration and ensure timely step-up transfers to the ICU [7]. Additionally, while HDUs have fewer staff than ICUs, they have the advantage of continuous patient vital sign monitoring [8]. The Early Warning Scoring System (EWSS), which integrates data obtained from continuous monitoring, may be valuable for the early detection of patient deterioration.

A study conducted in a trauma step-down unit demonstrated that the INDEX value (an AI-based EWS), which integrates multiple vital signs, often increased before the onset of overt clinical instability, highlighting the potential benefits of such alert systems in detecting early deterioration [18]. Delays in activating the rapid response system (RRS) have been shown to increase mortality rates [19]. Furthermore, a delay of four to six hours or more from the onset of physiological abnormalities to ICU transfer (i.e., delayed transfer) is associated with a higher risk of mortality [20].

Our data showed that NEWS could predict unexpected ICU admissions with good accuracy, as well as de novo vasopressor use and the composite outcome (either unexpected ICU admission, intubation, de novo vasopressor use, or de novo use of NPPV) with fair accuracy in the HDU setting. In a previous study, NEWS demonstrated a greater ability to identify patients at risk of combined outcomes such as cardiac arrest, unanticipated ICU admission, or death within 24 hours (AUC: 0.873; 95% CI: 0.866-0.879) in an inpatient setting [21]. NEWS has also been reported as a useful tool for predicting unplanned ICU transfers, cardiac arrests, and short-term mortality within 48 hours in both ER and prehospital settings [11,12]. In line with previous studies, our data confirm that NEWS has high predictive accuracy for unexpected ICU transfers in the HDU setting. However, the prediction efficacy of both NEWS and VSI for endotracheal intubation and de novo NPPV use was relatively low. This may be attributed to the small number of intubations in the HDU (n=6), and the exclusion of intubations performed after ICU transfer from the outcomes.

In Figure 3, the NEWS trend approaching outcomes did not significantly increase. In contrast, the VSI score demonstrated a significant upward trend, indicating that VSI was more sensitive to detecting deteriorating changes. In our study population, supplemental oxygen use, which is a component of NEWS but not VSI, was observed among 59.1% of vital sign sets. In NEWS, two points are assigned when supplemental oxygen is administered. About half (48.8%) of vital sign sets included impaired consciousness, defined as GCS≦14. When dementia or baseline disorientation is considered "non-alert," three points are automatically added to the NEWS score, potentially overestimating the total score. As most of our HDU population was admitted from the ER, distinguishing between new-onset confusion and pre-existing dementia can be challenging in real-world practice. These factors likely explain why NEWS was higher upon HDU admission and did not exhibit a significant trend toward score elevation as outcomes approached, compared to VSI. In this study, based on ROC analysis, the appropriate threshold for NEWS ranged between 6.0 and 8.0. Given that the original threshold for high-risk NEWS is seven points, our findings suggest that this threshold is an appropriate cutoff value for detecting adverse outcomes. In a previous study, the high risk of NEWS (7≥ points) was strongly correlated with 30-day mortality, with a correlation coefficient of 0.95 in the Japanese RRS registry study [22].

In our study, NEWS was used instead of the latest version, NEWS2. Since NEWS2 includes subjective and additional respiratory information, such as new-onset altered consciousness and chronic CO_2_ retention status [23], gathering this information retrospectively can be challenging.

Although VSI demonstrated modest predictive efficacy for unexpected ICU transfers, intubation, and de novo vasopressor use, it was significantly less effective than NEWS in predicting the composite outcome. One prospective, single-center, before-and-after study found that patients monitored with VSI in a step-down unit experienced significantly shorter durations of cardio-respiratory instability and fewer episodes of serious and persistent instability [18,24].

Using a high-risk threshold of VSI≧3.0, the proportion of patients identified as high risk was 20.7%, even among those with outcomes. However, in our ROC analysis, the optimal threshold for VSI was found to be 1.5. It suggests that a cutoff of 1.5, typically considered low risk, may be more appropriate for identifying high-risk patients in the HDU setting. From time trend analysis, lowering the threshold to VSI≧1.5 for high-risk led to a significant increase in the identification of patients with outcomes from 36% to 84% at about two hours before the first outcome (Figure 4). This suggests that, despite the traditional high-risk threshold of VSI≧3.0, a lower cutoff like VSI≧1.5 may better identify high-risk patients. VSI incorporates five parameters, whereas NEWS uses seven, notably including changes in consciousness and the administration of supplemental oxygen. Since VSI does not account for supplemental oxygen use or alterations in consciousness, there is a risk that it may underestimate a patient’s condition compared to NEWS. Specifically, when supplemental oxygen is administered, it can affect vital signs such as respiratory rate and SpO_2_, leading to a potentially lower VSI score, even though the patient’s condition may be more severe than reflected by the score.

In our study, 59.1% of vital sign sets included supplemental oxygen use, which may have contributed to VSI underestimating the severity of their condition. If VSI were adjusted, like NEWS, to account for oxygen use, it could more accurately reflect patient severity and become a more reliable scoring system in the future. On the other hand, NEWS requires manual input for oxygen administration and changes in consciousness, which cannot be automatically extracted from telemetry monitoring systems. This means registered nurses must assess these factors and manually record them on the vital sign flow sheet. Furthermore, while the NEWS protocol suggests adjusting the frequency of vital sign monitoring based on risk level, it has been reported that failure to adjust monitoring frequency occurred in about half of the cases, and such protocol violations were associated with increased 30-day mortality [25].

In contrast, VSI relies on five objective vital signs, allowing for automatic calculation, which likely made it easier to track consistent, time-based changes. This makes VSI a more feasible tool in HDUs, as it can reduce the workload on medical staff by utilizing telemetry monitoring for automatic scoring. Additionally, VSI showed a significant upward trend in relation to patient outcomes over time. In this retrospective observational study, the frequency of vital sign measurements did not increase when VSI levels rose. According to the protocol, if an alert system were implemented to recommend more frequent monitoring as EWS rises [13,14], high-risk patients could be identified more accurately. Our findings suggest that EWS, including NEWS and VSI, may be a valuable tool for predicting patient deterioration in HDUs. However, further prospective, multi-center studies are needed to validate these results.

Limitations

Our study has several limitations. First, the study period was relatively short (three months), the sample size was limited (356 participants), and the number of cases for certain outcomes (endotracheal intubation, de novo use of NPPV) was small, which may have affected the reliability of the results. Second, as a single-center observational study, its applicability to other HDUs remains uncertain, and prospective, multi-center studies are required to confirm its generalizability. Third, 1,150 out of 4,621 vital sign sets were excluded due to missing data, underscoring the need for more consistent and accurate vital sign monitoring, even within an HDU setting. Fourth, the assessment of consciousness was subjective, relying on the Glasgow Coma Scale (GCS). This complicated the distinction between new alterations in consciousness and pre-existing cognitive impairment, potentially leading to overestimating the NEWS score.

## Conclusions

This study showed that NEWS and VSI are useful for predicting acute deterioration in patients in the HDU. In particular, NEWS is effective for predicting ICU admission and using vasopressor agents, and VSI is a promising tool for capturing early changes. However, the accuracy of predicting the use of tracheal intubation and NPPV was low, so future research should seek to improve the prediction of these outcomes. As this is a retrospective study at a single facility, a prospective multi-center study is needed to verify its reproducibility at other facilities.
